# *Drosophila* blood cell chemotaxis^☆^

**DOI:** 10.1016/j.ceb.2014.04.002

**Published:** 2014-10

**Authors:** Iwan Robert Evans, Will Wood

**Affiliations:** 1 Faculty of Medical and Veterinary Sciences, University of Bristol, University Walk, Bristol BS8 1TD, UK; 2 Department of Infection and Immunity, The Medical School, University of Sheffield, Beech Hill Road, Sheffield S10 2RX, UK; 3 The Bateson Centre, University of Sheffield, Sheffield S10 2TN, UK

## Abstract

*Drosophila melanogaster* contains a population of blood cells called hemocytes that represent the functional equivalent of vertebrate macrophages. These cells undergo directed migrations to disperse during development and reach sites of tissue damage or altered self. These chemotactic behaviors are controlled by the expression of PDGF/Vegf-related ligands in developing embryos and local production of hydrogen peroxide at wounds. Recent work reveals that many molecules important in vertebrate cell motility, including integrins, formins, Ena/VASP proteins and the SCAR/WAVE complex, have a conserved function in these innate immune cells. The use of this model organism has elucidated how damage signals are activated by calcium signaling during inflammation and that the steroid hormone ecdysone activates immune competence at key developmental stages.

## Highlights

• Integrin-mediated adhesion used by *Drosophila* blood cells to migrate *in vivo*.
• SCAR/WAVE is required for lamellipodia but also for clearance of apoptotic cells.
• The formins Fhos and Diaphanous regulate *Drosophila* macrophage migration and morphology.
• Calcium waves drive hydrogen peroxide production to regulate inflammatory migrations.
• The steroid hormone Ecdysone controls the onset of immune competence.

**Current Opinion in Cell Biology** 2014, **30**:1–8

This review comes from a themed issue on **Cell adhesion and migration**

Edited by **Anna Huttenlocher** and **Erik Sahai**

For a complete overview see the Issue and the Editorial

Available online 8th May 2014

0955-0674/$ – see front matter, © 2014 The Authors. Published by Elsevier Ltd. All rights reserved.

**http://dx.doi.org/10.1016/j.ceb.2014.04.002**

## Introduction

Chemotaxis is the directed movement of cells (or an organism) towards or away from a chemical source. A classical example of chemotaxis is the movement of immune cells, such as neutrophils or macrophages, towards chemoattractants released at sites of infection or injury (*e.g.* fMLP and CSF-1) [1]. This process has been studied intensively *in vitro*, while the slime mould *Dictyostelium discoideum* has also proven vital in dissecting out the migration machinery and its regulation [2]. Whilst understanding regulation of cell migration represents a key biological problem, the fact that so many studies focus on immune cell motility reflects the diverse range of human diseases driven or exacerbated by inflammation.

Insects contain a population of blood cells, called hemocytes (Box 1), which make up the cellular component of their innate immune system [3, 4]. Given the genetic tractability and imaging capabilities of *Drosophila melanogaster*, the hemocytes of this organism have emerged as a prime cell type with which to study the regulation of migration and inflammation *in vivo*. Hemocytes are functionally equivalent to vertebrate macrophages and undergo chemotaxis to undergo developmental migrations and reach sites of tissue damage, while also detecting and removing apoptotic cells, debris and pathogens [4]. In this review we will discuss recent developments in our understanding of the machinery used by *Drosophila* hemocytes to chemotax during both developmental and pathological events occurring over the lifespan of a fruit fly. We will also focus on the latest work elucidating how damage signals are triggered and immune cell activation controlled.

Box 1 **Blood cell lineages in Drosophila**

*Drosophila* fruit flies contain a population of blood cells called hemocytes that consists of at least three cell types: plasmatocytes, lamellocytes and crystal cells. Plasmatocytes are migratory, phagocytic and resemble vertebrate macrophages; lamellocytes are induced during immune responses to encapsulate invading parasites with their large lamellar processes [65]; crystal cells are non-motile and rupture during immune responses to activate the phenoloxidase pathway and the melanization cascade [66], a humoral form of host defense. Insect blood cells have been used extensively as a model for blood cell specification and proliferation, since many of the signaling pathways used during vertebrate hematopoiesis are conserved and related transcription factors employed [67, 68], such as the GATA factor Serpent [69] and the RUNX homologue Lozenge, which is specifically required for the production of crystal cells [70]. Embryonic hemocytes are derived from the head mesoderm [71], while a second wave of hematopoiesis occurs in the lymph gland, with cells released from this organ during larval stages [72]. Migration studies typically focus on the highly motile plasmatocytes, which disperse over the entire embryo during the course of development [71]. Plasmatocytes persist through to adult stages [72] and are often referred to simply as hemocytes (as we have done in this review) or macrophages.

## Hemocytes use an evolutionarily conserved migration machinery to undergo chemotaxis

**Figure 1:**
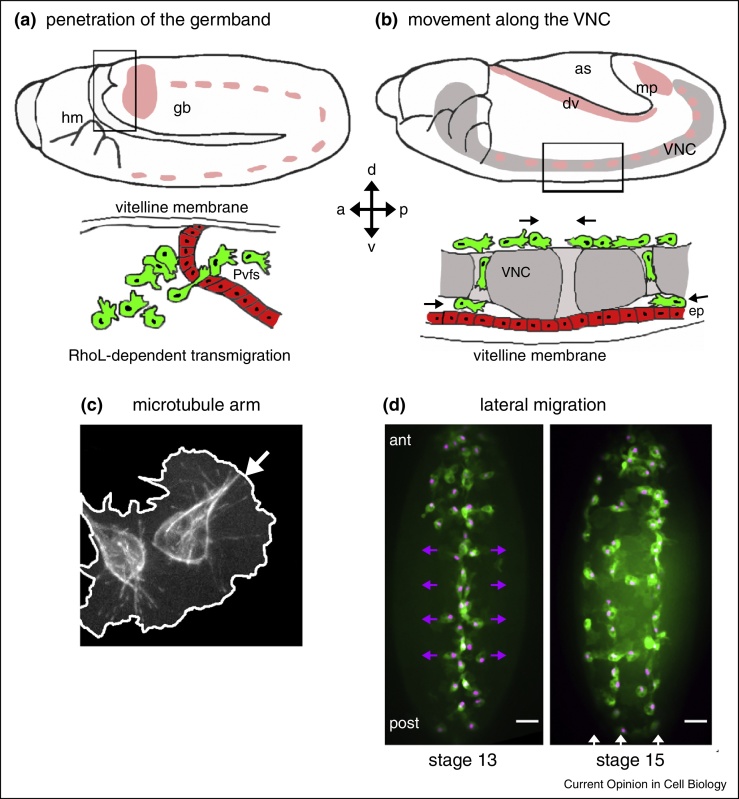
Embryonic migration routes and chemoattractant expression. Schematics showing expression of Pvf2 and Pvf3 chemoattractants (pink shading) in the developing *Drosophila* embryo at stages 11 **(a)** and 12 **(b)**. Cartoons below embryos correspond to boxed regions and show RhoL-dependent invasion of the germband (gb) towards a source of Pvfs, some of which is expressed by the developing malphigian tubules (mp) (a) and movement along the developing ventral nerve cord (VNC; grey) (b); arrows indicate hemocyte movements at these stages of development. During progression along the VNC hemocytes are tightly confined between the ventral side of the VNC and epithelium (ep) and as they migrate along the VNC in response to the Pvf ligands that are expressed there, the epithelium and VNC separate, creating a channel for hemocyte progression. Hemocytes also migrate along the developing dorsal vessel at this stage (dv); a = anterior, p = posterior, d = dorsal, v = ventral, lat = lateral. Later in development cell–cell repulsion begins to occur and this depends upon the microtubules, which are frequently bundled into an arm-like structure (arrow) that facilitates persistent migration **(c)**. Microtubules labeled *via* Clip-GFP expression in hemocytes; white line indicates edges of hemocytes, drawn according to mCherry-moesin localization (not shown). After initial dispersal hemocytes migrate at right angles from the ventral midline to the edges of the VNC (purple arrows) to form three lines (white arrows) on the ventral side of the embryo, immediately beneath the epithelium **(d)**. Maximum projection images show GFP and nls-red stinger localization in hemocytes from the ventral side of the embryo; scale bars represent 50 μm; ant = anterior, post = posterior.

Hemocytes migrate as individual cells tightly confined between tissues when colonizing the embryo (Figure 1a,b) [5, 6]. Dispersal is critical for normal morphogenesis [7, 8, 9, 10, 11], allowing hemocytes to reach distant locations where their developmental functions are necessary and facilitates surveillance against potential pathogens. Consequently, dispersal is a carefully orchestrated and hard-wired process and its stereotyped nature provides numerous opportunities at which to determine the genetic requirements for chemotaxis. After dispersal, hemocytes become responsive to wound stimuli owing to downregulation of developmental cues [12], suggesting a prioritization of developmental cues over wound cues; a large overlap exists in the machinery used to respond to either cue. Migrating hemocytes possess large actin-rich lamellipodia into which microtubules protrude from the cell body. These microtubules are often bundled into an ‘arm-like’ structure (Figure 1c), which facilitates persistent motility [13]. A number of classic cytoskeletal regulators are autonomously required within hemocytes for dispersal or normal motility, including the GTPases Rho, Rac and Cdc42 [14, 15], and actin regulators Ena [6] and fascin [16, 17••]; all these play related roles in vertebrate cells.

A family of PDGF/Vegf-related ligands called the Pvfs is expressed along the routes hemocytes take through the embryo (Figure 1a,b) [18, 19], suggesting they operate as chemoattractants to drive dispersal. Pvf signaling *via* the receptor Pvr is indispensible for both hemocyte viability and migration [18, 20, 21]. Importantly, blocking hemocyte apoptosis in *pvr* mutants rescues hemocyte numbers in the embryo [20], but fails to restore developmental dispersal fully [19, 20], while misexpression of Pvf2 can re-route hemocytes [5, 18], signifying Pvr promotes more than simply hemocyte survival. The route most sensitive to loss of Pvr signaling is penetration of the extended germband: here invasive hemocytes breach an epithelial barrier, involving a hemocyte-dependent disassembly of epithelial adhesions (Figure 1a; [22]). This strongly resembles transepithelial migration of vertebrate immune cells and critically depends on a small GTPase, RhoL [22]. RhoL function during transepithelial migration depends upon Rap1, which itself operates upstream of integrins in both hemocytes [23] and transmigrating vertebrate leukocytes [24]. Recent work has demonstrated that the main β-integrin (encoded by *myospheroid*) is required for normal hemocyte motility and migration to wounds in both embryos and pupae [25••, 26•]. In *myospheroid* embryos failed separation of the ventral nerve cord (VNC) and epithelium [25^••^] contributes to dispersal phenotypes. Loss of ECM (*laminin*) [27] or integrin complex components (*rhea*/*talin* and *fermitin 1*) also impairs migration [25••, 26•]. Whilst loss of integrin complex components did not interfere with repolarization towards wounds [25••, 26•], microtubule dynamics within hemocytes were affected with rapid and repeated collapse of microtubule arms observed *in vivo* [25^••^], presumably explaining the defects in contact inhibition of motility (cell–cell repulsion — a phenomenon that depends on microtubules [13]) observed in *myospheroid* mutants. Collapse events may occur *via* uncoupling of the actin and microtubule cytoskeletons or increased actin retrograde flow forcing microtubules rearwards when integrin-mediated anchoring of actin to ECM is absent.

## Nucleation of actin filaments in migrating hemocytes

Although *Drosophila* cell RNAi screens identified numerous regulators of cellular morphology and the actin cytoskeleton [28, 29, 30], *in vivo* roles for many regulators have not been investigated. Addressing how hemocytes generate actin networks to drive migration has provided novel insights into hemocyte function *in vivo*.

*SCAR* encodes the *Drosophila* homologue of the WAVE proteins, activators of the Arp2/3 complex. *SCAR* interacts genetically with *pvr* during hemocyte dispersal along the VNC [5], potentially becoming activated downstream of Pvr *via* Vav, a Rac GEF downstream of Pvr in border cell migration [31^•^], or the adapter Pico/Lamellipodin [32^••^]. Unsurprisingly *SCAR* is necessary for all hemocyte migrations and drives formation of lamellipodia, revealing that branched Arp2/3-nucleated actin is a key component of these protrusive structures *in vivo*. However loss of SCAR also leads to hemocytes becoming engorged with undigested apoptotic cells [33^••^], a phenotype possibly related to *SCAR* mutant trafficking defects previously only observed in *Dictyostelium* [34]. Remarkably, blocking apoptosis to remove the source of apoptotic cells rescues hemocyte lamellipodia and dispersal and also partially restores their motility, suggesting that SCAR-independent mechanisms to form lamellipodia exist and that these can be suppressed by contact with apoptotic cells [33^••^], which may have important implications for regulation of macrophage behaviors following contact with apoptotic cells in disease situations. *SCAR* was also recently shown to be necessary for the migration of pupal macrophages to wounds [35^•^].

Formins represent another means to nucleate actin filaments. *Drosophila* contain examples of seven of the eight human formin families [36] and the homologue of the formin FHOD (encoded by *fhos*/*knittrig*) localizes to the rear of migrating pupal hemocytes and is required for spreading of pupal macrophages *in vitro* and normal migration to wounds [37^••^]. FHOD formins are thought to be activated by Rho kinase/Rock [36] and this seems to be the case for Fhos [37^••^]; Fhos may therefore act as a Rock-dependent effector of the RhoA-mediated retraction events necessary during migration to wounds [15]. How the activities of the numerous actin regulators known to operate in hemocytes are integrated to facilitate coordinated cell migration will doubtlessly be an important area to watch. Notably the group of Mark Peifer recently showed that Ena antagonizes Diaphanous (in both hemocytes and epithelial cells), which helps control the nature of filopodial protrusions ultimately produced [38^•^].

## Developmental dispersal after downregulation of the Pvfs

After migrating the length of the ventral midline, hemocytes undergo a characteristic migration to the edges of the VNC ([19]; Figure 1d). These movements correlate with downregulation of the Pvfs at the midline. Overexpression of Pvfs along the midline delays lateral migration [19], suggesting loss of attractive ligands controls the timing of this event. Mathematical modeling of hemocyte movements raises the intriguing possibility that contact inhibition explains this patterning: simulations of hemocytes released from the ventral midline reproduce lateral migration patterns seen in embryos [39^••^]. Importantly, reducing hemocyte numbers, a key parameter in the model, causes this pattern to break down both *in vivo* and in simulations [39^••^]. The underlying molecular basis for repulsion remains to be established, but potentially targets microtubules, since depolymerization or hyperstabilization of microtubules or loss of the microtubule-binding protein Clasp/Orbit inhibits contact inhibition [13]; dynamic microtubules are also needed during contact inhibition between fibroblasts *in vitro* [40]. As hemocytes cluster together at wound sites and other sites of pathology, mechanisms to override repulsion must exist to enable normal macrophage behavior. Later in development a subpopulation of hemocytes closely associates with the larval peripheral nervous system, establishing a hematopoietic niche [41^••^]. Physical disturbance of these hemocytes results in their re-homing to this niche [41^••^], suggesting the presence of attractive signals regulating developmental migrations post-embryogenesis.

## Regulation of migration to sites of pathology

As cells of the innate immune system, the primary role of hemocytes is host defense against invading pathogens and altered self. Hemocytes therefore localize to sites of tissue damage, cancerous growth and cell death (epithelial wounds [15, 42, 43], *RasV12;scribble*^−/−^ clones [44, 45] and loser cells resulting from cell competition [46], respectively). Tissue resident hemocytes also become activated to deal with damage and promote recovery independent of migration (*e.g.* in UV-irradiated eye discs; [47^•^]).

**Figure 2:**
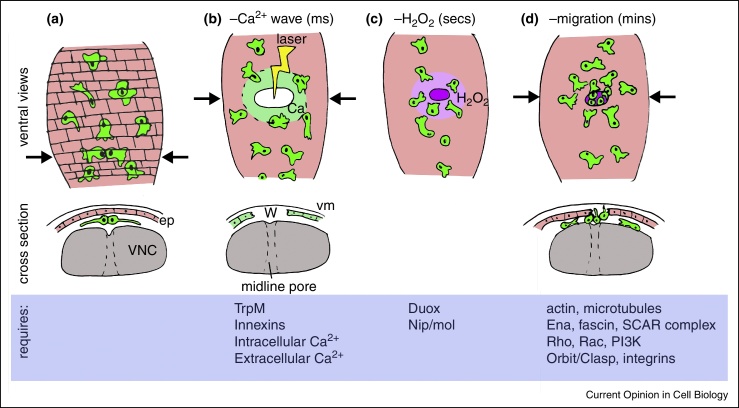
Calcium waves direct inflammatory migration of hemocytes. Ventral and cross-sectional views (anterior-posterior position indicated by arrows) showing immune cell recruitment to sites of tissue damage in *Drosophila* embryos. Hemocytes (green) sit immediately beneath the epidermis (ep, pink) on the ventral nerve cord (VNC, grey) **(a)**. Laser wounding of the epithelium causes an almost instantaneous calcium wave to flood through the epithelium *via* cell–cell junctions **(b)**; this depends upon functional cell–cell junctions and TrpM. An increase in intracellular calcium activates the NADPH oxidase Duox *via* its EF hands driving hydrogen peroxide production **(c)**. Hydrogen peroxide is necessary for the recruitment of hemocytes to this point of tissue damage, which is an active, migratory process requiring the function of the actin and microtubule cytoskeletons **(d)**. The relative timescale is indicated in brackets.

**Figure 3:**
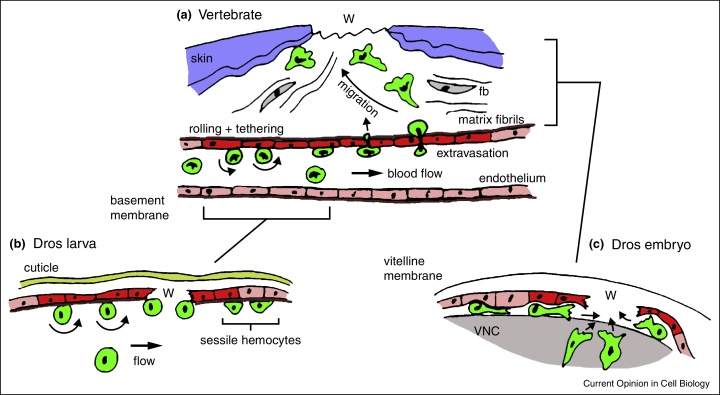
Comparison of migration to wounds in larval and embryonic stages of *Drosophila* development with vertebrate inflammatory responses. Cartoon of macrophage migration to wounds in vertebrates **(a)**. Macrophages (green) form transient adhesions with activated endothelial cells (red) and roll, leading to arrest and extravasation and penetration through the basement membrane (brown) before migrating though tissue largely composed of fibroblasts (fb) and ECM to reach wound sites (W). Larval hemocyte responses **(b)** consist of an adhesive capture that recapitulates rolling and tethering of vertebrate leukocytes; sessile hemocytes do not respond to wounds. Migration of hemocytes to wounds in the embryo occurs in the context of an environment containing ECM deposited between closely opposed tissues (epithelium and VNC) and requires active migration and resembles movement of vertebrate leukocytes post-extravasation.

The embryonic wound response is perhaps the best-characterized example of hemocyte chemotaxis; here hemocytes rapidly repolarize and migrate to sites of damage (Figure 2, Figure 3). As is the case following tail fin wounds in zebrafish larvae [48], the NADPH oxidase Duox becomes activated, leading to the production of hydrogen peroxide at wound sites. Duox is both necessary and sufficient for hemocyte recruitment [12, 49••]. In worms, flies and fish wounding induces a rapid calcium flash through the epithelium [49••, 50, 51••], which, in flies at least, leads to Duox activation *via* a pair of calcium-sensing EF hands in an intracellular loop (Figure 2) [49^••^]. How hemocytes decode the hydrogen peroxide wound cue is not known, but the zebrafish Src family kinase Lyn contains a conserved cysteine residue, oxidation of which regulates Lyn activity and is necessary for neutrophil chemotaxis to hydrogen peroxide and wounds [52]. This cysteine is conserved in Src42A in flies [52], suggesting this mechanism may be conserved through evolution, although Src42A has an anti-inflammatory role limiting epithelial cell responses to damage in flies [53].

PI3K signaling is specifically required for hemocyte wound responses in embryos, leading to the hypothesis that inflammatory responses could be regulated *via* G-protein coupled receptors, similar to other chemoattractants [19]. Alternatively PI3K signaling might be involved in hemocyte activation (*i.e.* a priming event rendering hemocytes competent to respond to wounds). Curiously PI3Kγ contributes to the wandering migration of neutrophils in zebrafish [54], but appears dispensable for hemocyte developmental migrations [19].

## Adhesive capture and hemocyte activation

During late embryogenesis, the primitive fly heart begins to beat and hemocytes are pumped around internal spaces as a constituent of the insect blood for the rest of the lifecycle, although some hemocytes remain attached to the epithelium in sessile patches. From larval stages onwards hemocytes are captured from the circulation *via* adhesion, with no contribution from the sessile population [42]. This ‘adhesive capture’ superficially resembles the rolling and tethering of vertebrate leukocytes ahead of their extravasation; embryonic migration more closely resembles chemotaxis of macrophages through connective tissue after extravasation (Figure 3). In pupae sessile patch hemocytes recommence motility and become wound responsive [43]. The signals driving inflammatory migration in larvae and pupae remain uncharacterized, but as wounding of the latter triggers integrin-dependent migration and epithelial calcium waves [26•, 55••], a similar mechanism to that of the embryo may be employed.

In larvae and adults activation of adhesion may facilitate capture: the typical blood cell response to damage and infection in Lepidopteran insects (the order containing moths and butterflies) is adhesion, which can be mediated *via* cytokine-like molecules such as plasmatocyte spreading peptide (PSP) [56, 57]. Injection of PSP into lepidopterans removes immune cells from circulation, presumably *via* adhesion to internal tissues [56]. Likewise, hemocyte chemotactic peptide (HCP) facilitates recruitment to wounds in moth larvae and directs chemotaxis of their blood cells *in vitro* [58]. Therefore systemic release of similar molecules may activate *Drosophila* hemocytes to enable capture at wounds. Recruitment to other sites of pathology (*e.g.* tumors) post-embryogenesis is also likely to occur *via* adhesive capture from circulation. Whether local infections can trigger focal recruitment of hemocytes remains unclear — chemotaxis towards pathogens is yet to be demonstrated. Homing of hemocytes to tumors is associated with damage or degradation of the basement membrane [45], which might expose adhesive signals or activate hemocytes to become adherent. Indeed, activation may represent the key step controlling immune responses. The steroid hormone ecdysone has long been associated with control of *Drosophila* development [59] and two recent studies have confirmed ecdysone to stimulate hemocyte motility, and its crucial role activating clearance of apoptotic cells and immune surveillance during metamorphosis [60••, 61••]. The transition back to a more classical migratory chemotaxis to wounds correlates with the beginning of metamorphosis and is prevented by expression of dominant negative ecdysone receptor in hemocytes [60^••^]. Ecdysone also turns on immune responses in embryos, since treatment with ecdysone analogues is sufficient to activate immune competence ahead of schedule [62^••^]. Rac1 and Basket/JNK signaling have also been previously implicated in hemocyte activation [63] and therefore represent potential downstream targets of signaling pathways to trigger recruitment of hemocytes from the circulation.

## Conclusions

Hemocytes have long been investigated as part of the innate immune responses to systemic infection [64], but have recently received substantial interest as a model cell type to understand the regulation of cell migration in the context of an intact and immune competent organism. We are now beginning to have a more complete understanding of the molecular mechanisms used by these highly migratory cells to reach the locations necessary for their range of functions and needed for their responses to pathology. As researchers fill in the gaps in our knowledge, we anticipate hemocytes will become a prime cell type to probe regulation of signal integration *in vivo* and the challenge for *Drosophila* researchers will be to use the powerful genetics available in the fly to identify novel targets involved in these processes.

## Competing interests statement

The authors declare no competing financial interests.

## References and recommended reading

Papers of particular interest, published within the period of review, have been highlighted as:

• of special interest
•• of outstanding interest
